# Clinical Characteristics and Outcomes of Very Old Critically Ill Patients in a Portuguese ICU: A Retrospective Cohort Study

**DOI:** 10.7759/cureus.103970

**Published:** 2026-02-20

**Authors:** Ines Pinto Pereira, Ana Tojal, Raquel Torres, Vasco Silva, Beatriz Vieira, Margarida Gonçalves, Mariana Teixeira, Paulo Marçal, Paula Fernandes

**Affiliations:** 1 Critical Care Medicine, Unidade Local de Saúde Gaia/Espinho, Vila Nova de Gaia, PRT; 2 Intensive Care Unit, Unidade Local de Saúde Gaia/Espinho, Vila Nova de Gaia, PRT

**Keywords:** critical care, critical illness, elderly, frailty, icu, mortality, very old patients

## Abstract

Introduction

Patients aged ≥80 years represent a growing subgroup of intensive care unit admissions and frequently present with a high burden of multimorbidity and frailty. Frailty has consistently been associated with increased short- and long-term mortality in this population, often demonstrating stronger prognostic value than chronological age alone. However, data on long-term outcomes among frail very old patients in Portuguese intensive care units remain limited, particularly in the context of an aging population and potential resource constraints.

Methods

We conducted a single-center retrospective cohort study including all patients aged 80 years or older admitted to a polyvalent intensive care unit in Portugal between 2021 and 2023. Data collected included demographic characteristics, comorbidities assessed using the Charlson Comorbidity Index, number of daily prescription drugs, and frailty status assessed with the Clinical Frailty Scale, with patients classified as frail or non-frail. Illness severity at admission was evaluated using the Simplified Acute Physiology Score II, the Acute Physiology and Chronic Health Evaluation II score, and the Sequential Organ Failure Assessment score. Outcomes included the use of organ support therapies, decisions regarding life-sustaining treatment, lengths of stay in the intensive care unit and hospital, and survival at intensive care unit discharge, hospital discharge, and at one month, six months, and one year after discharge.

Results

Among 3,359 admissions, 328 (9.8%) patients were very old. The mean age was 83.9 years; 58.1% were male, and 35.1% were frail. Frail patients had a higher comorbidity burden (p<0.00001), greater medication use (p = 0.0019), and presented with higher Acute Physiology and Chronic Health Evaluation II scores, while Sequential Organ Failure Assessment and Simplified Acute Physiology Score II values were similar between groups. The use of organ support therapies and lengths of stay in the intensive care unit and hospital did not differ significantly. Intensive care unit and hospital mortality were comparable between frail and non-frail patients; however, long-term survival differed significantly, with lower one-year survival among frail patients compared with non-frail patients (47.0% versus 62.0%, p = 0.009).

Conclusions

Frailty was common among very old patients admitted to intensive care and, in unadjusted analyses, was associated with worse one-year survival, despite similar short-term mortality and resource utilization. Routine assessment of frailty may improve prognostication, support shared decision-making regarding life-sustaining treatments, and inform post-intensive care follow-up strategies tailored to this highly vulnerable population.

## Introduction

Over recent decades, rising life expectancy has led to a steady increase in the number of very old patients (VOPs), defined as individuals aged 80 years or older, admitted to intensive care units (ICUs). This demographic shift has intensified debate regarding the appropriateness of ICU admission and treatment in this population, given the close association between advanced age and a higher burden of multimorbidity, frailty, functional dependence, and cognitive impairment, which together represent a major challenge for contemporary intensive care practice [1-3].

Evidence from large multinational European cohort studies has shown that frailty, rather than chronological age alone, is an independent predictor of adverse outcomes among VOPs admitted to the ICU. The VIP1 study, which included 5,021 VOPs, reported that frailty, defined as a Clinical Frailty Scale (CFS) [4] score greater than four (i.e., CFS ≥5), was present in 43.1% of participants and was independently associated with increased 30-day mortality after ICU admission, with a hazard ratio of 1.54 (95% CI 1.38-1.73) [1].

The VIP2 study, focusing on acutely admitted VOPs, similarly demonstrated that frailty assessed using the CFS was an independent predictor of 30-day survival after ICU admission. Importantly, the addition of comorbidity burden, cognitive decline, or disability measures did not improve prognostic performance beyond frailty assessment alone, reinforcing the central role of frailty in outcome prediction in this population [2].

Data from Portuguese ICUs remain limited. A recent national cohort including patients aged 65 years or older reported a substantial prevalence of frailty and worse short-term outcomes among frail individuals, including higher illness severity at admission, greater use of organ support therapies, and increased ICU and hospital mortality [3]. However, only 24.8% of patients in that cohort were VOPs, and follow-up was largely restricted to in-hospital outcomes, limiting the generalizability of those findings to VOPs and to longer-term trajectories.

Despite the growing representation of VOPs in ICUs, important gaps remain regarding prognostic determinants and long-term outcomes after critical illness in this population. Most available studies focus primarily on immediate or 30-day mortality, while outcomes beyond hospital discharge remain insufficiently characterized [5,6]. Improved knowledge of longer-term survival is essential to inform ICU admission decisions, support shared decision-making with patients and families, and guide planning of post-ICU care.

The present single-center retrospective cohort study was designed to address these gaps by evaluating both short- and long-term outcomes in VOPs admitted to a Portuguese ICU over a three-year period. Specifically, we aimed to characterize baseline comorbidity burden and frailty status, reasons for ICU admission, illness severity, use of organ support therapies, and decisions regarding life-sustaining treatment, as well as survival at ICU discharge, hospital discharge, and at one month, six months, and one year after discharge. To our knowledge, this is the first study conducted in a Portuguese ICU focusing exclusively on VOPs with extended follow-up beyond hospital discharge.

## Materials and methods

Study design and setting

We conducted a single-center retrospective cohort study including all consecutive patients aged 80 years or older admitted to the polyvalent intensive care unit of the Gaia and Espinho Local Health Unit (Unidade Local de Saúde Gaia e Espinho), Portugal, between January 2021 and December 2023. No exclusion criteria were applied beyond the age threshold.

Data collection

Data were extracted from electronic medical records and included demographic characteristics (age and sex), comorbidities assessed using the Charlson Comorbidity Index (CCI) [7], and the number of chronic daily prescription medications prior to hospital admission.

Frailty was assessed using the Clinical Frailty Scale (CFS) [4], reflecting patients’ baseline functional status prior to acute illness. CFS scores were assigned by six trained investigators based on information documented in the electronic medical record at the time of ICU admission. When descriptive information regarding baseline functional status was available, investigators classified patients according to standardized CFS criteria. When a CFS score had already been documented at admission without detailed functional descriptors, the recorded value was retained. If insufficient information was available to determine frailty status, the CFS was considered missing. Formal inter-rater reliability testing was not performed.

Based on the CFS, patients were classified as frail (CFS ≥5) or non-frail (CFS <5), in accordance with previous studies involving very old ICU patients [1,2].

Illness severity at ICU admission was assessed using the Simplified Acute Physiology Score II (SAPS II) [8], the Acute Physiology and Chronic Health Evaluation II (APACHE II) [9], and the Sequential Organ Failure Assessment (SOFA) score [10].

Outcomes

The primary outcomes were ICU and hospital mortality. Secondary outcomes included the use of organ support therapies, namely invasive and non-invasive mechanical ventilation, vasopressor support, and renal replacement therapy, as well as the performance of tracheostomy. Evolution of organ dysfunction was assessed using the delta SOFA score, calculated as the difference between discharge and admission values and computed only in ICU survivors to avoid distortion related to terminal events and treatment withdrawal. Decisions to withhold or withdraw life-sustaining treatment and the length of stay in the ICU and hospital were also evaluated.

Long-term outcomes included survival at one month, six months, and one year after hospital discharge. Vital status was determined through review of electronic medical records and consultation of the Portuguese National Health Service User Registry (Registo Nacional de Utentes), which allows verification of survival status. No patients were lost to follow-up at any of the predefined time points.

Statistical analysis

Missing data were minimal (<5% for all variables). Descriptive statistics were used to characterize the study population and are reported as means with standard deviation (SD) for normally distributed variables, medians with interquartile range (IQR) for non-normally distributed variables, and frequencies and percentages for categorical variables.

Comparisons between frail and non-frail patients were performed using Pearson's chi-square test for categorical variables. When expected cell counts were less than five, Fisher's exact test was used instead. Continuous variables were compared using an independent-samples Student’s t-test when normally distributed and the Mann-Whitney U test when normality assumptions were not met. Normality was assessed using the Shapiro-Wilk test and visual inspection of histograms. All statistical tests were two-tailed, and a p-value below 0.05 was considered statistically significant. Statistical analyses were performed using IBM SPSS Statistics, version 29.0.1.0 (IBM Corp., Armonk, New York, USA).

Ethical considerations

The study was approved by the local Ethics Committee. No direct or indirect patient identifiers were collected, precluding individual traceability. All procedures were conducted in accordance with the Declaration of Helsinki, the European Union General Data Protection Regulation, and applicable national legislation. The requirement for informed consent was waived due to the retrospective nature of the study.

## Results

Study population and baseline characteristics

Baseline demographic characteristics, comorbidity burden, number of chronic daily prescription medications prior to hospital admission, and frailty status are summarized in Table 1.

**Table 1:** Baseline characteristics of VOPs. Baseline demographic characteristics, comorbidity burden, number of chronic daily prescription medications prior to hospital admission, and frailty status. Data are presented as n (%), mean ± SD, median (IQR), or range, as appropriate. VOPs: very old patients; SD: standard deviation; IQR: interquartile range.

Variable	VOPs (n = 328)
VOPs admitted, n (% of all patients)	328 (9.8)
Sex	
Male, n (%)	190 (57.9)
Female, n (%)	138 (42.1)
Age (years)	
Age, mean ± SD	83.9 ± 3.3
Age, median (IQR)	83 (81–86)
Age, range	80–93
≥90 years, n (%)	18 (5.5)
Charlson Comorbidity Index (CCI)	
CCI, mean ± SD	6.2 ± 2.1
CCI, median (IQR)	6 (5–7)
CCI, range	4–14
Chronic daily prescription medications	
Number of drugs, mean ± SD	6.6 ± 3.4
Number of drugs, median (IQR)	6 (4–8)
Number of drugs, range	0–17
Clinical Frailty Scale (CFS)	
Non-frail (CFS 1–4), n (%)	213 (64.9)
1–Very fit	1 (0.3)
2–Fit	12 (3.7)
3–Managing well	115 (35.1)
4–Living with very mild frailty	85 (25.9)
Frail (CFS 5–8), n (%)	115 (35.1)
5–Mild frailty	48 (14.6)
6–Moderate frailty	47 (14.3)
7–Severe frailty	16 (4.9)
8–Very severe frailty	4 (1.2)

During the study period, 3,359 patients were admitted to the ICU, of whom 328 (9.8%) met criteria for VOPs. Nine patients had more than one ICU admission during the study period; only the first admission was included in the analysis.

Among VOPs, male patients accounted for 57.9%; the mean age was 83.9 years (SD 3.3), with a median of 83 years (IQR 81-86) and a range of 80-93 years. Patients aged 90 years or older accounted for 5.5% of the cohort.

Overall, the comorbidity burden was moderate, with a mean CCI of 6.2 (SD 2.1) and a median value of 6 (IQR 5-7), ranging from four to 14. Daily medication burden was notable, with a mean of 6.6 (SD 3.4) and a median of six medications per day (IQR 4-8).

Frailty, assessed using the Clinical Frailty Scale (CFS), was present in 115 patients (35.1%), while the remaining 213 patients (64.9%) were classified as non-frail. Among frail VOPs, mild and moderate frailty were the most frequent categories, whereas severe and very severe frailty were less common.

Admission diagnosis and severity of illness

The distribution of admission diagnoses and severity scores at ICU admission is presented in Table 2. 

**Table 2:** Admission diagnoses and severity scores of VOPs. Distribution of admission diagnoses and severity scores among very old patients admitted to the intensive care unit. Admission diagnoses are presented as n (%). Severity scores are presented as mean ± standard deviation, median with interquartile range, and range, as appropriate. VOPs: very old patients; SD: standard deviation; IQR: interquartile range; SOFA: Sequential Organ Failure Assessment; APACHE II: Acute Physiology and Chronic Health Evaluation II; SAPS II: Simplified Acute Physiology Score II.

Variable	VOPs (n = 328)
Admission diagnosis, n (%)	
Ischemic stroke	62 (18.9)
Post-scheduled procedure monitoring	49 (14.9)
Septic shock	46 (14.0)
Trauma	41 (12.5)
Acute respiratory failure	29 (8.8)
Hemorrhagic shock	17 (5.2)
Heart failure	17 (5.2)
Hemorrhagic stroke/subarachnoid hemorrhage	16 (4.9)
Post-cardiac arrest	9 (2.7)
Other	42 (12.8)
Severity scores	
SOFA, mean ± SD	4.2 ± 3.5
SOFA, median (IQR)	3 (1–6)
SOFA, range	0–17
APACHE II, mean ± SD	18.1 ± 8.4
APACHE II, median (IQR)	16 (12–23)
APACHE II, range	6–42
SAPS II, mean ± SD	42.7 ± 16.0
SAPS II, median (IQR)	39 (30–53)
SAPS II, range	20–98

The most frequent admission diagnosis among VOPs was ischemic stroke (18.9%), followed by post-scheduled procedure monitoring (14.9%), septic shock (14.0%), and trauma (12.5%). Other causes of admission included acute respiratory failure (including 11 cases with COVID-19 infection), hemorrhagic shock, heart failure, hemorrhagic stroke, subarachnoid hemorrhage, post-cardiac arrest status, and other miscellaneous conditions.

Illness severity at ICU admission was moderate overall. The mean SOFA score was 4.2 (SD 3.5), with a median value of 3 (IQR 1-6) and a range from 0 to 17. The mean APACHE II score was 18.1 (SD 8.4), with a median of 16 (IQR 12-23) and values ranging from 6 to 42. The mean SAPS II score was 42.7 (SD 16.0), with a median of 39 (IQR 30-53) and a range from 20 to 98.

Comparison between non-frail and frail very old patients (VOPs)

Comparisons between non-frail and frail VOPs are detailed in Table 3.

**Table 3:** Comparison between non-frail and frail VOPs. Comparison of baseline characteristics, type of admission, illness severity, organ support requirements, evolution of organ dysfunction, length of stay, decisions regarding life-sustaining treatment, and mortality and survival outcomes between non-frail and frail very old patients. Continuous variables are expressed as median (IQR) and were compared using the Mann–Whitney U test. Categorical variables are expressed as n (%) and were compared using Pearson's chi-square test or Fisher's exact test, as appropriate. Odds ratios (OR) with 95% confidence intervals (CI) are presented for binary outcomes and were calculated for frail versus non-frail patients. VOPs: very old patients; IQR: interquartile range; SOFA: Sequential Organ Failure Assessment; APACHE II: Acute Physiology and Chronic Health Evaluation II; SAPS II: Simplified Acute Physiology Score II; ICU: intensive care unit; LOS: length of stay; RRT: renal replacement therapy; OR: odds ratio; CI: confidence interval.

Variable	Non-frail	Frail	Test	p-value	OR (95% CI)
Baseline characteristics					
Age, median (IQR)	83 (82–86)	83 (82–85)	U = 11367.5	p = 0.2846	-
Charlson Comorbidity Index, median (IQR)	5 (4–7)	6 (5–8)	U = 8376	p < 0.0001	-
Daily prescription drugs, median (IQR)	6 (4–8)	7 (5–9)	U = 9662.5	p = 0.0019	-
Type of admission			χ² = 2.3272	p = 0.3124	
Medical, n (%)	102 (47.9)	47 (40.9)			
Emergency surgical, n (%)	78 (36.6)	52 (45.2)			
Elective surgical, n (%)	33 (15.5)	16 (13.9)			
Severity scores					
SOFA, median (IQR)	3 (1–6)	3 (2–6)	U = 10808.5	p = 0.1179	-
APACHE II, median (IQR)	16 (12–22)	19 (12–25)	U = 10474.5	p = 0.0305	-
SAPS II, median (IQR)	38 (29–52)	40 (30–57)	U = 10794.5	p = 0.2068	-
Organ support					
Invasive ventilation, n (%)	69 (32.4)	37 (32.2)	χ^2 ^= 0.0017	p = 0.9675	0.99 (0.61–1.61)
Non-invasive ventilation, n (%)	27 (12.7)	22 (19.1)	χ^2 ^= 2.4483	p = 0.1177	1.63 (0.88–3.04)
Ventilator-free days, median (IQR)	2 (1–3)	1 (1–3)	U = 11080.5	p = 0.1560	-
Tracheostomy procedure, n (%)	5 (2.3)	4 (3.5)	Fisher's exact test	p = 0.7235	1.5 (0.39–5.64)
Vasopressor support, n (%)	68 (31.9)	37 (32.2)	χ^2 ^= 0.0021	p = 0.9630	1.01 (0.63–1.63)
Vasopressor-free days, median (IQR)	2 (1–4)	2 (1–3)	U = 1059	p = 0.1835	-
Renal replacement therapy, n (%)	7 (3.3)	9 (7.8)	Fisher's exact test	p = 0.0940	2.50 (0.90–6.96)
Renal replacement therapy days, median (IQR)	2 (1–2)	5 (3–5)	U = 19.5	p = 0.2225	-
Organ dysfunction evolution					
Delta SOFA, median (IQR)	-1 (-2–0)	-0.5 (-2–0)	U = 8156.5	p = 0.9601	-
Length of stay					
ICU, median (IQR)	3 (2–5)	3 (1–5)	U = 11486	p = 0.3524	-
Hospital, median (IQR)	7 (2–17)	9 (3–20)	U = 11229.5	p = 0.2150	-
Decisions about life-sustaining treatment					
Withholding decision, n (%)	34 (15.9)	23 (20.0)	χ^2 ^= 0.8479	p = 0.3572	1.31 (0.73–2.36)
Withdrawing decision, n (%)	28 (13.1)	16 (13.9)	χ^2 ^= 0.0379	p = 0.8457	1.07 (0.55–2.10)
Mortality/survival					
ICU mortality, %	14.6	21.8	χ^2 ^= 2.7231	p = 0.0989	1.63 (0.91–2.93)
Hospital mortality, %	25.4	27.8	χ^2 ^= 0.2363	p = 0.6299	1.14 (0.68–1.89)
One-month survival, %	71.8	65.2	χ^2 ^= 1.5413	p = 0.2144	1.36 (0.84–2.21)
Six-month survival, %	65.7	56.5	χ^2 ^= 2.7004	p = 0.1003	1.48 (0.93–2.35)
One-year survival, %	62.0	47.0	χ^2 ^= 6.8583	p = 0.0088	1.84 (1.16–2.91)

Age at admission was similar between groups. Frail patients had a significantly higher comorbidity burden, with a higher median CCI of six compared with five in non-frail patients (p < 0.0001) and a greater number of daily prescribed drugs, with median values of seven versus six (p = 0.0019).

Frail VOPs also presented with slightly higher illness severity at admission, reflected by a higher median APACHE II score of 19 compared with 16 in non-frail patients (p = 0.0305). Median SOFA and SAPS II scores did not differ significantly between groups.

The distribution of admission type (medical, emergency surgical, or elective surgical) was similar between groups (medical admissions: 47.9% in non-frail vs 40.9% in frail patients; p = 0.3124).

Organ support use was largely comparable between non-frail and frail VOPs, as shown in Table 3. Use of invasive mechanical ventilation was similar between groups, being required in 32.4% of non-frail patients and 32.2% of frail patients. Non-invasive ventilation was used in 12.7% of non-frail patients and 19.1% of frail patients, without reaching statistical significance. The number of ventilator-free days was comparable between groups. Tracheostomy was infrequently performed, occurring in 2.3% of non-frail patients and 3.5% of frail patients.

Vasopressor support was common in both groups, being required in 31.9% of non-frail patients and 32.2% of frail patients, with similar vasopressor-free days. Renal replacement therapy was infrequently required and did not differ significantly between groups, although numerically more frequent among frail patients (7.8% vs 3.3%).

The evolution of organ dysfunction was similar in both groups. The median delta SOFA was −1 in non-frail VOPs and −0.5 in frail VOPs. ICU and hospital length of stay were similar between non-frail and frail VOPs. Decisions regarding life-sustaining treatment were evenly distributed between groups, with no significant differences in withholding or withdrawal of treatment, as summarized in Table 3.

Regarding primary outcomes, ICU mortality was numerically higher among frail than non-frail VOPs, at 21.8% versus 14.6%, although this difference did not reach statistical significance. Hospital mortality was similar between groups, at 27.8% in frail patients and 25.4% in non-frail patients.

Survival at one month and six months after discharge did not differ significantly between groups, although survival was consistently numerically lower among frail patients. At one year after discharge, frail VOPs had significantly lower survival than non-frail patients (47.0% versus 62.0%; OR 1.84, 95% CI 1.16-2.91, p = 0.0088).

## Discussion

In this single-center cohort of very old patients (VOPs) admitted to the ICU, frailty was common and, in unadjusted analyses, was associated with worse long-term outcomes, despite broadly similar acute management and short-term outcomes between frail and non-frail individuals. Frail VOPs presented with a higher comorbidity burden, greater medication load, and slightly higher illness severity at admission. Nevertheless, ICU and hospital mortality were comparable between groups, whereas one-year survival was substantially lower in frail patients. These findings suggest that the impact of frailty may become more evident beyond the acute phase of critical illness.

Our results are consistent with evidence showing that frailty is a stronger determinant of outcome in ICU VOPs than chronological age alone, as demonstrated in large European multicenter cohorts such as VIP1 and VIP2 [1,2]. In those studies, higher Clinical Frailty Scale (CFS) scores independently predicted short-term mortality after adjustment for age, comorbidities, and severity scores. Although multivariable modeling was not performed in the present study, and therefore independent associations cannot be inferred, our findings extend prior observations by suggesting that the survival disadvantage associated with frailty may persist up to 12 months after discharge. This reinforces previous work identifying premorbid functional status, comorbidities, and frailty as key predictors of six-month and one-year mortality in elderly ICU populations [11,12].

The mortality and survival rates observed in this cohort fall within the wide ranges reported in systematic reviews and observational studies of older ICU patients, in which one-year mortality varies from approximately one-third to more than two-thirds of cases, depending on age distribution, case mix, admission criteria, and follow-up duration [5,11-13]. In this context, one-year survival rates of 62.0% in non-frail and 47.0% in frail VOPs indicate that a substantial proportion of VOPs can survive the first year after ICU admission, particularly when frailty is absent or mild. At the same time, the 15-percentage-point absolute difference between groups underscores the prognostic relevance of frailty and its importance in discussions regarding prognosis and post-ICU care planning. The trend toward higher ICU mortality among frail patients, although not statistically significant, is directionally consistent with the long-term findings and may reflect limited statistical power to detect modest early differences.

A notable observation is the apparent dissociation between ICU treatment intensity and long-term outcomes. Use of organ support therapies--including invasive and non-invasive mechanical ventilation, vasopressors, and renal replacement therapy--as well as ICU and hospital length of stay and evolution of organ dysfunction, were broadly similar between frail and non-frail VOPs. Frail patients did not appear to receive systematically less organ support or have shorter ICU stays. This suggests that poorer one-year survival among frail patients may be more closely related to underlying biological vulnerability and reduced physiological reserve rather than differences in access to or intensity of ICU interventions.

These results also complement existing data on illness severity scores in VOPs. Frail patients had higher APACHE II scores at admission, whereas SOFA and SAPS II scores were similar between groups. This pattern is consistent with prior observations that traditional severity scores retain prognostic value but do not fully capture the impact of premorbid function, frailty, and comorbidity burden [1,2,6,11,14-16]. The combination of comparable SOFA scores with significantly lower one-year survival in frail patients suggests that acute organ dysfunction alone does not fully explain outcomes and supports the concept that chronic vulnerability interacts with acute illness to shape long-term trajectories. This reinforces calls to integrate frailty and premorbid status into prognostic models and clinical decision-making for elderly ICU patients.

Analysis of life-sustaining treatment decisions suggests that frailty did not systematically lead to more frequent treatment limitation, as withholding and withdrawal decisions occurred at similar rates in frail and non-frail patients. This pattern indicates that decisions were likely based on comprehensive clinical and ethical assessment rather than on CFS scores alone. However, the absence of a clear gradient in treatment limitation despite marked differences in one-year survival raises the question of whether frailty could be more explicitly incorporated into shared decision-making processes to better align care intensity with long-term prognosis and patient preferences.

Tracheostomy was infrequently performed in both groups, and the study was underpowered to draw firm conclusions regarding its association with frailty. The low overall rate is consistent with cautious use of prolonged life-sustaining interventions in VOPs, for whom post-ICU recovery may be particularly complex. Larger studies are needed to better understand how frailty and baseline function influence decisions regarding prolonged mechanical ventilation and anticipated quality of life.

Taken together, these findings have several practical implications. First, they support routine frailty assessment using a simple tool, such as the CFS, at or before ICU admission, not as a rigid gatekeeping criterion, but as a means to inform discussions with patients and families about prognosis, expected trajectories, and the balance between survival and functional outcomes. Second, the broadly similar short-term mortality and resource utilization between frail and non-frail VOPs suggest that, once admitted, VOPs tend to receive intensive treatment irrespective of frailty status, emphasizing the importance of careful pre-ICU triage and early goals-of-care discussions when long-term survival and recovery may be limited.

In health systems such as the Portuguese National Health Service, where population aging coexists with constrained ICU capacity [17,18], these results underscore the need for integrated care pathways linking ICU care with ward-based management, rehabilitation services, and community follow-up. The substantial survival disadvantage associated with frailty suggests that structured post-ICU follow-up may be particularly valuable for frail VOPs, with a focus on early identification of functional decline, cognitive impairment, and caregiver burden, as well as timely access to rehabilitation or palliative and supportive care when appropriate.

Long-term survival was determined by reviewing electronic medical records and consulting the Portuguese National Health Service User Registry (Registo Nacional de Utentes), which allowed verification of vital status at predefined follow-up time points. No patients were lost to follow-up at one month, six months, or one year, reinforcing the completeness of survival data. However, because exact dates of death were not consistently available, survival was assessed at fixed intervals rather than using time-to-event methods, such as Kaplan-Meier curves or Cox regression, which may have limited a more granular evaluation of survival trajectories.

The study period overlapped with the COVID-19 pandemic, and 11 patients were admitted with COVID-19 infection. Although this represents a small proportion of the cohort, pandemic-related changes in case mix, ICU organization, and admission patterns may have influenced outcomes and should be considered when interpreting the findings.

This study has several strengths, including the use of contemporary real-world data from a Portuguese ICU, a setting underrepresented in the literature; the inclusion of all consecutive patients aged 80 years or older over a three-year period; the use of the CFS, a validated frailty measure that facilitates comparison with international cohorts; and 12-month follow-up, allowing a more comprehensive assessment of long-term survival.

Several limitations must be acknowledged. The retrospective single-center design carries inherent risks of information bias and residual confounding. As a multivariable adjustment was not performed, the association between frailty and one-year survival should be interpreted as unadjusted. Frail patients presented with higher comorbidity burden and greater illness severity at admission, which may partially account for differences in long-term outcomes. Therefore, residual confounding cannot be excluded. Frailty assessment was conducted retrospectively based on clinical documentation and by multiple investigators without formal inter-rater reliability testing, and some degree of misclassification bias cannot be excluded. Additionally, it is possible that the most severely frail individuals were not admitted to the ICU due to pre-existing treatment limitations or triage decisions, which may have attenuated differences in short-term mortality between groups. Finally, the sample size may have limited statistical power to detect modest differences in some outcomes, including ICU mortality, tracheostomy use, and specific organ support modalities.

Future research should aim to validate these findings in larger multicenter cohorts using prospective designs with standardized assessment of frailty, cognition, and functional status at baseline and follow-up. Integrating frailty measures with traditional severity and comorbidity scores may improve prognostic models and help identify subgroups of VOPs with distinct risk profiles and care needs. Further studies should also explore whether the prognostic impact of frailty varies according to illness severity or type of admission, and should incorporate patient-centered outcomes, including functional recovery, quality of life, and caregiver burden. Ultimately, incorporating frailty into both prognostic assessment and shared decision-making may help deliver more individualized and value-congruent care to VOPs at the threshold of intensive care.

## Conclusions

VOPs represented a small but clinically relevant proportion of ICU admissions in this Portuguese center and exhibited substantial baseline heterogeneity in comorbidity burden and frailty status. Frailty, as assessed by the CFS, was common and did not significantly influence ICU or hospital mortality or the use of organ support therapies, but in unadjusted analyses was associated with lower one-year survival, highlighting its relevance for long-term outcomes.

These findings support routine frailty assessment in VOPs considered for ICU admission, not as an exclusion criterion, but as an important component of prognostic evaluation, to inform shared decision-making regarding life-sustaining treatments and to guide post-ICU follow-up strategies tailored to individual vulnerability and goals of care.
